# Supplementary material for: Are carbon emissions trading and green financial instruments synergistic? -Comprehensive quantitative research based on content analysis
Source: PLoS One. 2024 Mar 7;19(3):e0298601. doi: 10.1371/journal.pone.0298601 (PMC10919601; doi:10.1371/journal.pone.0298601)
Supplement: S1 Data — (ZIP) [file pone.0298601.s001.zip › Data/地级市Z_F工作报告.zip!/╣π╢1⁄2/╔ε█┌╩╨/└┤╫╘╣1⁄2╓┌║┼í╛┬φ┐╦╩2╛▌═°í┐_2011.docx]

各位代表：

　　现在，我代表深圳市人民政府向大会作工作报告，请予审议，并请各位政协委员和其他列席人员提出意见。

　　一、"十一五"发展成就及2010年工作回顾

　　"十一五"期间，全市人民在市委的坚强领导下，以邓小平理论和"三个代表"重要思想为指导，全面贯彻落实科学发展观，有效应对国际金融危机的严重冲击，积极实施《珠江三角洲地区改革发展规划纲要》，较好地完成了"十一五"规划的主要目标任务，全市经济、政治、文化、社会和生态文明建设取得显著成就。

　　一是综合经济实力连续跃升。"十一五"期间，我市经济总量接连跨越五个"千亿"大关。2010年，预计实现本市生产总值9500亿元，比2005年增长86%。人均生产总值1.46万美元，增长58.9%。规模以上工业增加值、高新技术产品产值、地方财政一般预算收入和全口径财政收入跃上新台阶，分别为4050亿元、10176亿元、1106.8亿元和3507亿元，增长88%、108%、168%和158%。外贸出口2042亿美元，实现十八连冠。社会消费品零售总额3000亿元，实现翻番。五年累计完成全社会固定资产投资7740亿元，接近前25年总和。

　　二是经济竞争力显著增强。成为首个国家创新型城市。全社会研发投入占GDP比重达3.64%，是全国平均水平的两倍。核心技术自主创新能力得到提升，PCT国际专利申请量连续7年居国内首位，五年累计专利申请量和授权量分别达到19.38万件和10.67万件，基因研究、通信技术水平达到国际一流。国家超级计算深圳中心及电子信息产品标准化等5家国家工程实验室落户深圳，中科院深圳先进技术研究院、华大基因研究院建成运行。国家发改委批准在深设立国家基因库。产业结构明显优化，三次产业比例为0.1∶47.2∶52.7，实现了三次产业结构从"二三一"到"三二一"的战略性调整。现代产业体系框架基本形成，高新技术、金融、物流、文化四大支柱产业增加值占GDP的比重超过60%。战略性新兴产业迅速崛起，在全国率先出台实施生物、互联网、新能源三大产业振兴规划及产业政策。金融业增加值占GDP比重由8%提高到13%左右，国内金融机构本外币存、贷款余额双双实现翻番，分别达到20850亿元和15921亿元。创业板成功推出，深交所2010年IPO家数位居全球第一，深圳多层次资本市场更加完善。深圳港集装箱吞吐量和机场旅客吞吐量分别为2251万标箱和2671万人次，增长39%和64%。文化产业增加值占GDP比重达6.05%，文化与科技、金融、旅游融合发展的模式初步形成。总部经济发展迅速，在深投资的世界500强企业达到180家。经济效益持续提高，每平方公里产出GDP4.77亿元、税收1.74亿元，分别提高2.23亿元和1.07亿元。万元GDP能耗和水耗分别为0.51吨标准煤和20.3立方米，相当于全国平均水平的1/2和1/10。二氧化硫和化学需氧量排放量分别下降31%和37%，圆满完成国家节能减排任务。

　　三是城市功能明显提升。《珠江三角洲地区改革发展规划纲要》明确了我市国家综合配套改革试验区、全国经济中心城市、国家创新型城市、中国特色社会主义示范市和国际化城市"一区四市"的战略定位。城市总体规划获国务院批复，确定了未来十年的城市发展蓝图。地铁建设取得重大成果，总长178公里的1-5号线即将全面开通。"一横八纵"骨干路网基本建成，各组团交通联系更加紧密。广深港客运专线、厦深铁路等项目加快建设，进一步强化了深圳全国综合交通枢纽城市地位。一批水、电、油、气工程进展顺利，资源能源保障能力明显提升。城市更新加快推进，城中村综合整治和市容环境提升行动成效明显。五年新建各类公园435个，"公园之城"初步建成。环境保护从传统的治污保洁向生态建设和低碳发展转变，城市生活污水集中处理率从46%提升到85%，污水再生利用率从不足1%上升到27.1%，被确定为全国首批生态文明建设试点地区。

　　四是改革开放深入推进。国家正式批准我市为国家综合配套改革试验区，经济特区范围扩大到全市，前海深港现代服务业合作区发展上升为国家战略。行政体制改革稳步推进，设立光明、坪山新区，完成市政府大部门体制改革，在全国率先实施公务员分类管理和聘任制改革，事业单位改革取得阶段性成果。创新基层社区体制机制，社会工作制度初步建立，社会组织登记管理体制改革先行一步。人口管理模式取得突破，居住证制度全面推行。第三轮财政体制改革取得显著成效，财政资金管理运营水平进一步提高。市属国有企业三项制度改革基本完成，资产总额与利润总额实现倍增。推进公交特许经营改革，公交服务向规模化、专业化转变。"走出去"战略迈出坚实步伐，华为、中兴等龙头企业形成全球布局，中国-越南（深圳-海防）经贸合作区建设有序推进。深港西部通道、福田口岸等一批重大跨界基础设施投入使用，深港合作不断深化。深澳、深台合作加强。深莞惠一体化进程加快，"双转移"、"双到"工作扎实推进，服务全省的能力不断增强。积极做好对口帮扶工作，出色完成对口支援四川、甘肃地震灾区恢复重建任务，受到中央的高度肯定。援疆工作取得阶段性成果。

　　五是社会民生持续改善。人民生活水平明显提高，居民人均可支配收入达3.22万元，居内地大城市首位。城镇居民登记失业率始终控制在3%以内，零就业家庭户数保持动态归零。企业退休人员养老待遇大幅提高，社会保险覆盖面继续扩大，在全国率先实现全民医保。在明显缓解户籍低收入家庭住房困难的基础上，保障性安居工程的重点逐步转向各类人才和"夹心层"群体。社会救助、社会福利和优抚安置三大体系基本建立，连续四次被评为"全国双拥模范城"。

　　教育、卫生、文化、体育等社会事业加快发展。高标准实施免费义务教育，惠及学生272万人次。新增基础教育学位26万个，完成原特区外96所村办小学标准化改造。高等教育发展取得突破，在校学生人数增加2.2万人，清华、北大、哈工大深圳研究生院成为我市高等教育和自主创新的重要支撑，深圳大学新增博士学位点3个，深圳高等职业教育水平全国领先。市第三人民医院、疾控中心等17个重大卫生设施建成使用，全市医疗机构总数增长68.8%，医疗机构床位数增长33.6%。社康中心达到607家，实现全覆盖。一批重大公共文化设施投入使用，市属公益文化场馆在全国率先实行免费开放，城市街区24小时自助图书馆系统与"市民文化大讲堂"获国家"文化创新奖"。深圳被联合国教科文组织授予"设计之都"称号，连续两次被评为"全国文明城市"。成功申办第26届世界大学生夏季运动会，各项筹办工作全面展开。

　　社会安全形势总体保持稳定，重大恶性案件继续下降，市民群众安全感进一步提升。食品安全"五大工程"、"四大体系"初步建成，药品安全专项整治成效明显，成功应对了人禽流感、甲型H1N1流感等重大疫情。安全生产、应急管理、信访维稳工作扎实推进。军民共建工作积极开展。人口计生、统计、司法、档案、民族、宗教、外事、港澳、台务、侨务、民防、气象、防震减灾等各项工作取得新进步。

　　六是政府自身建设不断加强。认真执行法律法规和人大各项决议，自觉接受人大的法律监督、工作监督和人民政协、民主党派的民主监督及社会各方面的监督。市政府系统共办理代表重点建议11件、建议2877件，政协建议案19件、提案3184件，代表和委员满意和基本满意率达到99%以上。法治政府建设取得重大进展，制定全国第一个法治政府建设指标体系。开展第四轮行政审批制度改革，率先清理规范非行政许可审批和登记事项。加强行政电子监察，全面试行政府绩效管理。加快推进政务公开和电子政务建设，市行政服务大厅被评为全国"人民满意的公务员集体"。继续加大反腐倡廉力度，严肃查处了一批典型案件，依法惩处了一批腐败分子，廉政建设取得新进展。

　　2010年是深圳不平凡的一年，胡锦涛总书记亲自出席深圳经济特区建立30周年庆祝大会并发表重要讲话，赋予了特区新的历史使命，为深圳未来发展指明了方向。温家宝总理专程来深视察，对新形势下经济特区的发展作出了重要指示。新一届政府全面贯彻落实胡锦涛总书记重要讲话、温家宝总理重要指示精神和市第五次党代会、五届人大一次会议的决策部署，按照"五个继续、五个努力"的要求，弘扬"想干、敢干、快干"精神，推动经济社会平稳较快发展，为"十一五"画上了圆满的句号。

　　（一）科学谋划经济发展方式转变。召开加快转变经济发展方式专题会议，出台关于加快转变经济发展方式的决定及行动计划，推动经济进入科技引领、创新驱动、内生增长、低碳绿色的发展轨道。大力推进自主创新，引进光启高等理工研究院等一批高层次创新科研团队。制订新材料、文化创意两大产业振兴规划，实施战略性新兴产业重点项目计划，支持694个产业项目，2010年生物、互联网、新能源三大新兴产业增速分别为30%、24.2%和29.3%。国家低碳城市试点工作进展顺利，成立深圳排放权交易所，成功举办世界电动车大会，在全国率先投放使用纯电动出租车。认真组织编制全市"十二五"规划。

　　（二）全力推进经济平稳较快增长。市领导率队分赴300多家企业开展下基层送服务促转变调研活动，主动为企业排忧解难。着力扩大固定资产投资，6大领域80个项目集中开工，华星光电主体厂房提前封顶，长安标致项目前期工作有序推进。落实国家促进消费有关政策，积极扩大本地消费，进一步释放了居民消费潜力。大力实施内贸战略，举办或参加21个国内经贸洽谈会，深圳产品内销总量继续提升。进一步扩大对外贸易，优化外贸结构，一般贸易出口比重达31.3%，提高2个百分点。

　　（三）着力改善社会民生。认真落实全市社会建设工作大会精神，把社会建设摆在与经济建设同等重要位置。精心实施深圳经济特区建立30周年"十项民生工程"，办好发放医保补助等惠民实事。实施积极就业政策，促进4.9万名失业人员实现再就业，城镇登记失业率控制在2.45%以内。着力构建和谐劳动关系，劳动纠纷案件下降25.6%。出台关于推进教育改革发展率先实现教育现代化的决定，成为国家教育综合改革试点城市。启动"百校扶百校"工程，建成3所全寄宿制普高学校，新增普高学位9000个。深圳大学人事制度改革付诸实施，南方科技大学获准筹办，深港高等教育合作取得新进展。基本公共卫生服务均等化水平位居全国前列，社康中心全面实行国家基本药物"零加成"制度。成功举办联合国教科文组织创意城市网络大会，提升了深圳文化形象。积极构建立体防控体系，加强矛盾纠纷排查化解，深入推进严打整治行动，社会治安进一步好转。实施人才安居工程，启动安居型商品房建设，全面实施住房公积金制度。认真落实国务院关于坚决遏制房价过快上涨有关政策精神，出台严格的调控政策，努力保持房地产市场健康发展。实现基本养老保险关系转移接续的平稳过渡。积极落实国家稳定消费价格总水平有关政策，市场供应充足、秩序良好。初步完成第六次全市人口普查。

　　（四）加快推进特区一体化。出台特区一体化发展总体思路和工作方案，明确了一体化的目标、路径和步骤。经济特区现行113部法规和70部规章得到全面梳理，有效解决了特区一体化相关法规的适用问题。按照基础设施先行原则，加快推进南坪快速二期、丹平快速、沿江高速等重大交通项目建设，41公里地铁线路投入试运营。北线引水和东江水源二期工程相继通水，铁岗水库扩建工程完工移交，供水管网改造工程取得积极成效。基本实现原特区外100万户有线数字电视用户的整体转换。光明、坪山等功能区和龙华、大运等新城建设明显加快，有力地带动了原特区外地区的整体发展，实现了特区一体化当年初见成效的预期目标。

　　（五）全面优化城市环境。完善城市更新政策体系，积极推进大冲、岗厦、南科大校区等重点项目，实现了从局部改造到全面更新的跨越。市容环境提升行动取得突破性进展，街景美化、建筑物与公共设施外立面翻新、口岸环境和户外广告整治成效明显。大运中心、深交所大厦、京基100等一批标志性建筑封顶，107、205国道、北环大道等改造基本完成。加强环境治理，龙华、观澜污水处理厂二期项目开工。深入开展生态市建设，建成区域绿道335公里、城市和社区绿道77公里。

　　（六）继续深化改革开放。进一步推进公务员制度改革，公开招聘首批行政执法类公务员，新进公务员全部实行聘任制。设立前海管理局，探索法定机构运作新模式。加快户籍制度改革，推行积分入户政策，拓宽了外来务工人员入户渠道。全面加强深港合作，启动河套地区发展规划及工程研究，加快推进东部通道等跨界基础设施建设。以ECFA签订为契机，组团参加"台湾·广东周"活动，深台合作取得新进展。与汕尾共建深汕特别合作区，探索区域统筹发展新模式。

　　各位代表，这些成绩的取得，是党中央国务院、省委省政府和市委坚强领导的结果，是全市人民艰苦奋斗、努力拼搏的结果。在此，我代表深圳市人民政府，向奋斗在各条战线的全市广大干部群众和外地来深建设者，向给予我们不懈支持的人大代表、政协委员、各民主党派、工商联和社会各界人士，向中央和国家有关部门、中央和各省区市驻深单位，驻深解放军、武警官兵，向所有关心和支持深圳发展的港澳同胞、台湾同胞、海外侨胞和国际友人表示崇高的敬意和衷心的感谢！

　　在看到成绩的同时，我们也要清醒地认识到存在的困难和问题：一是资源环境约束趋紧，经济增长内生动力还不够强，经济效益需进一步提高。二是人口增长过快和人口结构性矛盾突出，增加了解决住房、教育、医疗、交通等社会民生问题的难度，加大了社会管理的压力。三是城市二元结构仍然突出，城市功能、生态环境和文明素质与国际先进城市还有差距，城市现代化国际化水平需进一步提升。四是重点领域和关键环节的改革难度加大，特区高速发展过程中遗留下来的一些历史问题需要通过深化改革来解决，改革的系统性、协调性、科学性需进一步增强。五是服务型、法治型政府建设与科学发展的要求相比还有差距，政府服务水平和工作效率需进一步提升。这些矛盾和问题事关全局，影响长远，需要我们采取更加有力的措施，努力加以克服和解决。

　　二、"十二五"基本思路

　　各位代表，"十二五"是深圳经济特区新时期的第一个五年。胡锦涛总书记在深圳经济特区建立30周年庆祝大会上的重要讲话明确指出，中央将一如既往支持经济特区大胆探索、先行先试、发挥作用，要求深圳继续当好推动科学发展、促进社会和谐的排头兵。国务院先后批准《珠江三角洲地区改革发展规划纲要》、《深圳市综合配套改革总体方案》、《前海深港现代服务业合作区总体发展规划》，同意将深圳经济特区扩大到全市范围，深圳正以崭新的风貌，踏上新30年的宏伟征程。

　　过去30年，深圳迅速崛起，创造了举世闻名的"深圳速度"，打造了全国领先的"效益深圳"，为迈向更高层次的发展奠定了坚实基础。现在，深圳已站在一个全新的历史起点上，要在新一轮发展中应对挑战、再创辉煌，就必须以新理念引领发展，以新标杆指引方向。这个新理念就是以质取胜，这个新标杆就是"深圳质量"。

　　"深圳质量"具有鲜明的科学发展内涵，蕴含继承发展的逻辑，反映主动竞争的自觉，体现全面协调的要求，指引破解难题的路径。"深圳质量"是"深圳速度"量变基础上的跃升，是"效益深圳"内涵和外延的扩展。我们要牢固树立崇尚质量、追求卓越的思维与理念，把速度、效益优势转化为质量优势，实现从速度优先向质量优先转变，从注重经济增长向更加注重经济社会全面发展转变，使经济更有效益、民生更为幸福、文化更具品位、城市更富魅力、生态更加美好，当好推动科学发展、促进社会和谐的排头兵。

　　——创造"深圳质量"，就是坚持以质取胜，追求更高的物质文明，使创新能力更强，产业结构更优，内生动力更足，国际竞争力一流，以"深圳质量"彰显经济特区新优势。

　　——创造"深圳质量"，就是坚持以人为本，追求更高的社会文明，使法制更加健全，社会更加和谐，人民更加幸福，以"深圳质量"展示中国特色社会主义优越性。

　　——创造"深圳质量"，就是坚持文化强市，追求更高的精神文明，使人的发展更全面，人民生活丰富多彩，社会风貌昂扬向上，以"深圳质量"塑造新时期的深圳精神。

　　——创造"深圳质量"，就是坚持内涵发展，追求更高的城市文明，使城市布局更加合理，功能更加完善，现代化国际化水平更高，以"深圳质量"跻身世界先进城市行列。

　　——创造"深圳质量"，就是坚持低碳理念，追求更高的生态文明，使资源消耗更小，环境代价更低，人与自然更加和谐，以"深圳质量"实现可持续发展能力领先全国。

　　"十二五"时期深圳的主要发展目标是：本市生产总值年均增长10%左右，2015年达到1.5万亿元，人均GDP达到2万美元。战略性新兴产业增加值占GDP比重达到20%，第三产业比重提高到60%。全社会研发支出占GDP比重达到4%。万元GDP能耗下降到0.47吨标准煤，万元GDP水耗下降20%，化学需氧量、氨氮、二氧化硫和氮氧化物排放量分别下降21%、22%、4%和3.5%。居民人均可支配收入达到4.9万元。城镇居民登记失业率控制在3%以内。

　　"十二五"时期，我们要全力贯彻中央、省委和市委"十二五"规划建议精神，积极落实我市加快转变经济发展方式和加强社会建设两个决定，用足用好"四个先行先试"的有利条件，围绕创造"深圳质量"，努力做到"六个着力"：

　　（一）以加快转变经济发展方式为主线，着力提升经济发展质量

　　--把自主创新作为转变经济发展方式的中心环节。深入实施自主创新主导战略，加快建设国家创新型城市，推动经济发展从要素驱动向创新驱动转变，科技进步贡献率不低于60%。突出源头创新，力争在新一代信息技术、生物、新能源等若干领域取得突破，形成一批国际领先的自主知识产权和技术标准，打造核心技术创新之城。大力吸引、集聚和共享全球创新资源，加快完善开放型区域创新体系，积极促进产学研合作，打造国际创新中心。力争到2015年，国家级工程（技术）研究中心、重点实验室、工程实验室和企业技术中心达到50家。加快科技创新体制机制改革，促进创新、创业、创优"三创合一"，推动科技创新与金融、商业、文化、管理创新有机互动的横向集成创新，打造全球创新成本低、创新收益高的先进城市。

　　——把推动产业结构战略性调整作为转变经济发展方式的主攻方向。着力打造"高、新、软、优"的现代产业体系。"高"，就是抓住"高端"，抢占现代产业制高点。坚持发展高端产业，鼓励企业参与核心技术攻关，引进尖端技术产业化项目，尽快形成高技术产业群；加快占据产业高端，引导企业向产业链的高增值环节、高技术含量领域延伸，向资本运营、研发设计、品牌专利、核心制造、销售服务等方向发展；大力发展总部经济，实现高端要素的持续聚集。"新"，就是扶持"新兴"，把握未来产业话语权。紧紧抓住代表未来发展方向和增长潜力的新技术、新产品、新产业，着眼长远、及早布局，集中力量扶持生物、互联网、新能源、新材料、文化创意等战略性新兴产业发展，打造新的经济增长点。"软"，就是推动"软化"，增强产业发展驱动力。加快发展知识经济、网络经济和服务经济，做强做大软件、创意等产业。推动第二产业向生产性服务业的演变升级。促进服务业对制造业的融合与渗透，通过虚拟空间发展实体经济，突破土地等硬资源的约束，使"软资源"的投入成为利润的主要源泉和产业发展的主要动力。"优"，就是坚持"优质"，提升产业国际竞争力。引进和培育一批优质产业，形成产业集聚效应，保持产业领先地位；大力实施优质龙头企业带动战略，重点支持各行业的优质企业在深发展壮大，带动产业创新发展；深入实施品牌战略，生产优质产品，创立国际名牌，着力提高深圳产品的美誉度。

　　——把增强经济内生动力作为转变经济发展方式的重要途径。适应国家扩大内需的战略安排与自身转型发展的要求，促进经济增长由出口拉动为主向消费、投资、出口协调拉动转变。以推进特区一体化、产业升级和民生改善为重点，扩大投资需求，五年投资累计超过1万亿元，实现投资规模和投资效益双提升。建设定位准确、布局合理的特色商圈，培育新的消费热点，充分发掘消费潜力。大力发展电子商务等新型消费业态，加快国家电子商务示范市建设，实现实体消费空间和虚拟消费空间的有机结合。实施内贸战略，提高深圳产品的国内市场占有率。巩固传统出口市场，开拓新兴出口市场，优化出口结构，实现由出口大市向出口强市转变。

　　——把建设人才高地作为转变经济发展方式的有力支撑。大力实施人才强市战略，瞄准国际一流创新人才，加快优质人才资源的战略性集聚。以完善政策、加大投入、打造载体、强化服务为重点，营造人尽其才、才尽其用的人才环境。大幅提升自主培养人才的能力，努力发挥深圳引才、育才、用才的综合优势，最大限度激发各类人才的创造活力和创业热情。高水平制订人才发展规划，大力实施"孔雀计划"等专才计划，为创造"深圳质量"提供强大的智力支撑。

　　（二）以改善公共服务为重点，着力提升社会发展质量

　　——提高居民收入和社会保障水平。推进收入分配制度改革，实现居民收入增长与经济发展基本同步、劳动报酬与劳动生产率提高基本同步。建立健全工资正常增长机制，稳步提高最低生活保障、最低工资标准。推动以创业带动就业，创建充分就业城市和国家级创业型城市。稳步扩大社会保险覆盖面，健全社会福利体系，完善综合性社会救助体系。五年筹集和新建保障性住房24万套，形成覆盖中低收入人群和各类人才的住房保障体系。

　　——提高民生福利。大力发展教育事业，力争率先成为全国教育现代化先进城市。推进义务教育优质均衡发展，新建一批义务教育阶段学校。普及高中阶段教育，新改扩建11所高中。加快发展高等教育，办好南方科技大学，丰富大学城办学层次，引进香港知名高校来深合作办学，高校在校生规模达到15万人。加大对学前教育的投入，实现合理布局和规范管理。发展职业教育，培养高层次技能型人才。基本完成公立医院改革，实施公共卫生机构分类管理，引导社会资源发展个性化高端医疗服务。推进基本医疗服务标准化和重大卫生项目建设，让每一个城市功能组团至少拥有一家三级医院。到2015年，新增床位1.5万张，千人医生数和千人病床数分别达到2.6人和3.4张。落实公交优先战略，实施交通需求管理，深化公交体制机制改革，加快形成以轨道交通为骨架、常规交通为网络的公交体系，到2015年，公交分担率达56%以上，有效解决交通拥堵问题。

　　——提高市民群众安全感。全面推进社会管理创新，率先形成科学有效的利益协调、诉求表达、矛盾调处和权益保障机制。构建和谐劳动关系，维护劳动者合法权益。实施"织网工程"，形成覆盖全社会的民心网、服务网和工作网。加强对流动人口、出租屋的管理，强化社会治安基础环境治理，建成社会治安立体防控体系，着力破解制约社会治安工作的源头性问题，实现治安形势明显好转。加强生产安全、交通安全、食品药品安全等工作，健全城市灾害预警预防体系，提高防灾减灾能力。

　　——提高城市文化内涵。充分挖掘深圳作为移民城市的文化潜力，建设独具特色和魅力的城市文化，提升城市软实力。推进社会主义核心价值体系建设，提升市民文明素质，以人的全面发展促进社会的全面进步。重点发展创意文化，建成具有国际影响力的创意之城和时尚之都。构建普惠型公共文化服务体系，基层文化设施覆盖率达到全国领先水平。举办高水平文化活动，创作高品质文化产品，发展群众体育、竞技体育和职业体育，以文化体育事业大发展大繁荣增强城市的凝聚力和影响力。

　　——提高全社会法治水平。坚持依法治市，充分用好经济特区立法的优势，加强制度建设，完善符合国际惯例和国际规则、有利于提升行政效率的法治环境，使法治成为深圳经济特区的新优势。强化规则意识，依法规范社会群体之间的关系，建立良好的社会秩序。大力弘扬法治精神，利用公益广告等媒介，在全社会倡导尊重法律、尊重规则的良好风尚。健全社会诚信体系，完善信用信息共享机制，建立失信惩戒制度和守信激励制度。

　　（三）以绿色低碳为导向，着力提升生态发展质量

　　--突出节能减排，降低资源环境代价。积极应对全球气候变化，抓住低碳发展的战略机遇，率先建立新的标准体系和竞争规则，抢占发展先机。制定低碳发展总体规划，实行严格的节能减排准入管理，重点推进工业、建筑和交通运输等领域的节能减排工作。把环境容量和污染物排放总量指标作为产业发展布局的重要依据，形成以环保促进发展方式转变的倒逼机制。加大研发投入，抢占低碳技术制高点。优化能源结构，发展新能源和可再生能源，建设绿色电网，到2015年，清洁能源发电装机容量达到750万千瓦以上。建设低碳型、节约型政府，引导全社会树立低碳理念，形成绿色生产生活方式，率先建成资源节约型、环境友好型城市。

　　——突出环境治理，改善城市人居环境。深入开展水环境和大气环境综合治理，全面完成深圳河、观澜河、龙岗河、坪山河干流综合整治，逐步修复河流生态系统，改善大气环境质量，率先实现垃圾全程分类收集处理，努力打造碧水蓝天的人居环境。到2015年，集中式饮用水源地水质达标率100%，中心城区污水处理率95%，空气质量优良天数360天以上，城市垃圾无害化处理率95%。

　　——突出生态建设，维护城市生态平衡。完善生态补偿机制，严格保护基本生态控制线，构建"四带六廊"生态安全体系，保障城市基本生态空间和生态功能。新建森林（郊野）公园11个、社区公园50个，建成绿道网2000公里，形成覆盖全市、彼此连通的自然生态系统、城市公园系统和慢行交通系统，让市民享受到更多的绿色福利。

　　（四）以推进特区一体化为突破口，着力提升城市发展质量

　　——优化布局。瞄准国际一流标准，全面优化城市布局，以中心城区为核心，形成"三轴两带多中心"的轴带组团结构。高起点、高标准规划开发前海、光明、坪山、龙华、大鹏半岛等重点区域，形成布局合理、特色鲜明、配套完备、生态和谐的功能区发展新格局，促进区域整体性开发、组团式发展。探索城市发展单元制度，完善城市规划实施手段。

　　——再造空间。加快城市更新，初步完成农村城市化历史遗留违法建筑和违法用地的处理，基本完成原特区内的城中村转型改造，以及原特区外主要地区的城中村整治。加大重大项目用地和重点开发区域的土地整备力度，到2015年，力争通过土地整备释放150平方公里建设用地。节约集约利用土地，力争用不超过总面积4%的土地增量推动60%的GDP增长。加强地下空间综合利用，挖掘空间资源潜力。

　　——完善功能。建设一批重大交通基础设施，到2015年，形成总长229公里的城市轨道网和"七横十三纵"干线路网，基本实现特区交通一体化目标。加快珠三角城际轨道、深中通道、深港机场联络快线建设，形成深港半小时和珠三角一小时交通圈，拓宽深圳对外联系的战略通道。完成广深港客运专线深圳段、厦深铁路建设，继续强化港口和机场的枢纽地位，将城市辐射范围拓展到泛珠三角乃至东南亚地区。继续推进水、电、油、气重大项目建设，加快构建多元化的资源能源保障体系。以三网融合为重点，加强信息资源的开发利用和信息网络的互联互通，积极推进物联网、云计算的研发和示范应用，实现信息化向经济社会和城市发展的全方位渗透，率先建成无线城市，着力打造智慧深圳，以高水平的信息化增强城市竞争力。

　　——提升品位。突出海滨城市特色，彰显现代城市品格。借鉴香港、新加坡等世界先进城市经验，塑造人性化、生态化和特色化的公共空间环境。加强重点区域、重点地段和标志性建筑的规划设计，打造城市亮点和建筑精品。新建一批标志性文化设施与旅游景点，加强历史文化资源的保护性开发，提升城市文化品位。深入开展市容环境提升行动，加强机场、口岸、车站等重点地段的综合整治，提高城市管理的精细化水平，打造更干净、更美丽、更有序的城市。

　　（五）着力深化改革创新，为创造"深圳质量"提供制度保障

　　——发挥市场作用，激发市场活力。充分发挥市场配置资源的基础性作用，建立反映市场供求关系、资源稀缺程度和环境损害成本的生产要素和资源价格形成机制。加快投融资体制改革，逐步扩大社会投资的领域和范围，释放全社会的投资潜力。探索建立高度城市化地区土地管理新模式，促进存量土地资源有效流转和优化配置。改革商事登记制度，降低市场准入门槛。逐步健全企业集体协商制度。推动集体股份合作公司建立现代企业制度。创新非营利性国有资产运营和监管体制，发挥好国有经济的先导性、基础性、公共性作用。

　　——推动基本公共服务均等化，激发社会活力。科学界定基本公共服务的范围及标准，率先建立起政府提供基本公共服务、市场提供个性化高端化服务的社会服务体系，不断提高公共服务质量。推进政府购买公共服务，鼓励和引导社会主体兴办社会事业，增加公共服务有效供给。积极发展社会组织，规范社会组织行为，更好地发挥其在社会建设中的重要作用。深入推进户籍制度改革，创新流动人口管理机制。

　　——优化行政体制机制，激发政府活力。完善大部门体制改革，探索精简行政层级。推进公务员分类管理和聘任制改革，创新事业单位内部运作机制，扩大法定机构试点范围。加强干部队伍建设，健全责任体系，建立激励机制，充分调动干部队伍的积极性。建立规章、规范性文件定期清理机制，动态规范行政审批和行政服务事项，提高行政效率和执行力。以服务对象为中心，优化服务流程，提升政府服务质量。推进权力运行程序化和公开透明，建设廉洁政府。

　　（六）着力扩大区域合作，为创造"深圳质量"拓展空间

　　——推动对外交流与合作，建设国际化城市。营造与国际接轨的商务环境，打造集聚国际要素资源的战略高地。深入实施"走出去"战略，鼓励本土企业在全球布局。利用中国-东盟自由贸易区平台，把东盟作为拓展新兴市场的重点区域，增强深圳经济国际影响力。加强对外友好交流，积极举办或承办大型国际性会议、展览、赛事和文化活动，提升深圳的国际知名度。

　　——推动深港融合发展，共同打造世界级都市圈。从国家战略高度，探索深港合作新机制新模式，把前海地区打造成深港合作先导区、体制机制创新区、现代服务业集聚区和结构调整引领区，为全国现代服务业创新发展提供示范。合作开发落马洲河套地区，推进跨界基础设施建设，促进要素更便利流动，全面加强深港创新圈建设和各领域合作交流，打造深港优质生活圈。不断拓宽领域，推进深澳、深台合作。

　　——推动更大范围的区域合作，构建多层次区域合作体系。实施外溢型发展战略，从更高层次、更大范围调动资源。发挥深圳经济中心城市作用，密切深莞惠合作，加强与珠江口西岸地区的联系，促进珠三角一体化发展。创新区域合作体制机制，推进深汕特别合作区建设。做好对口援建工作，更好地服务全省、服务全国。

　　三、2011年主要工作

　　2011年，国际国内经济环境更为复杂。一是国际经济格局深度调整，世界经济复苏缓慢曲折，不确定、不稳定性因素仍然较多，贸易保护主义普遍抬头，外需趋紧态势还将持续，对我市外向型经济影响较大。二是国内宏观经济环境趋紧，中央实施积极的财政政策和稳健的货币政策，增强了宏观调控的针对性、灵活性和有效性，深圳要保持经济平稳较快增长，需付出更大的努力。三是区域竞争日益加剧，全国改革发展呈现千帆竞发、百舸争流的态势，深圳必须进一步加快转变经济发展方式、优化经济结构。压力就是动力，只要我们切实增强忧患意识、责任意识和创新意识，把困难估计得更充分一些，把准备做得更扎实一些，积极应对，努力工作，就一定能够克服压力、战胜挑战，开创深圳未来发展的新局面。

　　今年是"十二五"的开局之年，也是深圳"大运年"。我们将认真贯彻中央十七届五中全会、中央经济工作会议、省委十届八次全会、市委五届五次、六次全会精神，全面实施"十二五"规划，紧紧围绕加快转型升级、建设幸福广东、创造"深圳质量"这个中心，进一步解放思想，扎实工作，着力加强经济和社会建设，努力向全市人民交出一份满意的答卷。

　　今年我市经济社会发展的主要目标是：本市生产总值增长10%左右，城镇居民登记失业率控制在3%以内，居民消费价格指数104左右，万元GDP能耗和水耗分别下降到0.501吨标准煤和19.2立方米，化学需氧量、氨氮、二氧化硫和氮氧化物排放总量分别下降5%、5%、1%和1%。为此，我们将大力做好以下重点工作：

　　（一）全力办好大运盛会

　　第26届世界大学生夏季运动会是深圳历史上最盛大的综合性体育赛事，也是深圳提升城市品质、打造城市精神、展示城市风采、改善社会民生的重要机遇。要突出"办赛事、办城市，新大运、新深圳"的主题，加快转变经济发展方式，提高深圳的竞争力；全面改善人居环境，提高深圳的魅力；加强对外宣传，提高深圳的国际影响力；激发干部群众干事创业的热情，提高深圳的凝聚力。要把办赛事与改善民生牢牢结合起来，把大运会办成一项全面深入的惠民工程。

　　要唱响"青春大运、文明城市、和谐家园"主旋律，做好城市亮化美化净化工作，确保市容环境焕然一新，让深圳因大运而更美丽。开展公共文明提升行动、市民讲外语活动，树立良好的社会风尚，充分展现东道主风采，让深圳因大运而更文明。精心宣传城市、营销城市，向世界展示深圳现代时尚、青春活力的城市形象，让深圳因大运而更精彩。

　　要认真学习北京奥运、上海世博、广州亚运的成功经验，按照平安大运、畅通大运、人文大运的要求，按时保质完成所有场馆和相关配套设施建设，策划组织好开闭幕式、火炬传递等重大活动，优化竞赛总体方案，精心做好市场开发、交通保障、安全保卫、宣传外事、气象保障、志愿者服务等工作，营造全民参与大运、奉献大运、服务大运的氛围，确保举办一届精彩、简约、有特色、有影响力的大运会。

　　（二）增强自主创新能力

　　加大源头创新力度，组织实施并积极参与国家重大科技专项和重点技术攻关，力争在核心电子器件、高端通用芯片、超大规模集成电路等战略领域实现一批核心技术突破。夯实创新基础，基本建成国家超级计算深圳中心，继续推动中科院深圳先进技术研究院、华大基因研究院、光启高等理工研究院、电子信息产品标准化实验室等创新平台建设，加快组建深圳国家高技术产业创新中心，规划建设大沙河创新走廊。集聚创新资源，力争全年新增国家、省、市级重点实验室、工程实验室、工程研究中心和企业技术中心40家。完善区域创新体系，全面落实深港创新圈三年行动计划，推进双边和多边国际科技合作，实现开放式创新及创新升级。办好第十三届高交会。

　　（三）提升产业层次能级

　　着力发展战略性新兴产业。认真落实有关战略性新兴产业振兴规划及产业政策，制定产业发展指导目录和产业链建设计划，完善扶持政策。积极培育战略性新兴产业百强企业，加快推进软件产业、生物医药、新能源等产业基地和园区建设。力争年内生物、互联网、新能源产业规模达到1990亿元。积极推进国家海洋经济发展试点示范市建设，打造现代农业生物育种创新示范区。

　　提升高技术制造业竞争力。针对重点产业链的高端和缺失环节，开展上下游产业链招商。大力发展以自主品牌和自主技术为主的电子信息、航天航空、数字装备等高技术产业，进一步提升高新技术企业在全球市场的竞争能力。加快推进光纤宽带网络、第三代移动通信网络等城市信息基础设施建设，试点部署第四代移动通信网络，创建云计算服务创新试点城市，推动新一代信息技术产业加快发展。

　　加快发展现代服务业。出台国家服务业综合改革试点方案实施计划，设立服务业发展引导资金。加大金融创新力度，加快全国金融改革创新综合试验区、保险创新发展试验区建设，积极推动跨境人民币结算，发展私募基金和风险投资，完善多层次资本市场。加快物流园区建设，促进高端物流发展。大力发展具有深圳特色的文化产业及产业集群，支持深圳文化产权交易所搭建文化产业发展综合服务平台，办好第七届文博会。以软件开发为突破口，着力培育和发展离岸外包业务，加快建设服务外包基地，创建中国软件名城。继续发展会展、旅游、邮轮游艇等高端服务业，加快大鹏半岛海滨旅游度假区建设。积极发展总部经济，吸引国内外企业集团总部、区域总部及各类功能中心入驻深圳，年内新引进总部企业8家。

　　促进传统优势产业转型升级。优化整合优势传统产业发展资源，以品牌化、集约化为方向，提高印刷、黄金珠宝、钟表、服装、模具等行业自主研发、设计和工艺水平。制定加工贸易转型升级方案，推动来料加工企业加快转型，淘汰落后产能，为高端产业腾出发展空间。

　　加大扶持中小企业力度。实施中小企业上市培育、融资、产业链配套、人才与创新、市场开拓五个专项计划，促进中小企业快速发展。以创业板、中小企业板为平台，拓宽中小企业融资渠道，力争年内在海内外上市的本市中小企业达到30家以上。支持民营经济创新发展，发挥民营经济创造"深圳质量"的重要作用。

　　（四）优化增长需求结构

　　推动投融资体制改革实现重大突破。整合政府资源，建立更加规范化的投融资体制。组建市级投融资平台，全方位、多渠道筹措城市建设资金。进一步清理限制社会投资的政策法规，切实放宽对民营经济的市场准入，鼓励和引导社会资本进入城市更新改造、基础设施建设及社会事业等领域，力争社会投资比重达到75%以上。

　　加快重大项目建设。完成地铁二期工程，启动7、9、11号线建设，推进6、8号线前期工作。加快广深港客运专线、厦深铁路、沿江高速、南坪快速二三期、坪盐通道、大铲湾二期等重大基础设施建设，确保机场二跑道投入使用。大力推进长安标致、比亚迪新能源汽车等重大产业项目建设，确保华星光电一期工程建成投产。积极推进岭澳核电站三期、抽水蓄能电站、国家成品油储备深圳油库、西气东输二线深圳段、天然气高压输配系统主体工程等能源保障基础设施建设。加快实施保障性住房、教育、卫生、文化及市政道路改造等重大民生项目。

　　积极开拓内外市场。完善扩大居民消费长效机制，大力发展电子商务，加强商业网点规划布局，促进消费信贷，发展假日经济，培育新的消费热点。充分发挥深圳品牌、创新和服务优势，加强国内市场推广和销售渠道优化工作，力争工业产品内销比重达到45%左右。巩固传统出口市场，开拓以东盟为重点的新兴市场。扩大一般贸易和服务贸易出口，优化外贸结构。完善重大贸易摩擦快速反应协调机制、贸易安全与产业损害预警机制，保护本土企业利益。

　　（五）提高城市宜居水平

　　加强生态城市建设。政府部门全面推行合同能源管理，带动全社会的节能减排工作。探索建立新上项目碳排放标准评价体系，着力发展低碳产业，把若干产业园区建成低碳示范区。探索建立环境容量有偿使用、碳排放权和排污权交易制度。加大水环境综合整治力度，加快污水处理厂及配套管网设施的建设改造。加强大气污染治理，对主要电厂推行降氮脱硝，推广应用国Ⅳ车用燃油。力争推广新能源汽车2011辆。加快推进东部垃圾焚烧厂等垃圾处理设施建设，提高城市生活垃圾、建筑废弃物无害化处理和综合化利用水平。全方位建设包括各类公园在内的绿地系统、湿地系统和城市立体绿化走廊，新建绿道300公里，争创国家首批生态园林城市。

　　加大城市更新和土地整备力度。出台城市更新办法实施细则，着力推进福田环CBD、笋岗-清水河、大沙河、华为科技城等重点地区的城市更新。制定土地整备若干意见，切实推进土地确权工作，完善土地整备体制机制。强力推进违法建筑的查处工作。

　　提高城市管理水平。以大运会举办为契机，立足解决城市管理的热点难点问题，有重点、分阶段地开展专项整治行动，促进市容环境全面提升。提高城市绿化水平，推进景观照明提升工程。深入开展新一轮城中村综合整治工作，基本解决城中村脏、乱、差的问题。加强市容环境管控力度，联合整治非法营运，营造良好市容秩序。

　　加快特区一体化步伐。安排政府投资337亿元用于特区一体化建设，占全市政府投资规模的75.4%。以新功能区、新城和重点片区为节点，以城市轨道交通、快速干道为网络，重点突破，点线带面，高标准推进特区一体化。进一步优化原特区外的产业和人口结构，加强社会管理和公共服务，提高治安水平，推进全市软环境的一体化。

　　（六）释放体制机制活力

　　启动并完成区级换届工作与区级政府机构改革，统筹区级党委、政府机构设置，深化完善大部门体制。按照权责一致、重心下移的原则，科学划分市区事权、财权。整合街道和社区资源，完善基层管理体制。深化公务员分类管理和聘任制改革，探索公务员跨部门交流机制。推进事业单位管理体制和运行机制创新，启动并完成区属事业单位分类改革。完成全市居委会换届工作，力争居委会全面实现直接选举。完善社会组织管理的政策法规，逐步建立社会组织直接登记制度。

　　实施第四轮市区财政体制改革，进一步理顺公共财政的投入与保障机制。探索开征房地产税和环境保护税。进一步完善国有企业法人治理结构，加大国有资本经营预算改革力度，深化市属国有企业领导人员选拔任用改革。加快水、电、气等资源价格改革，探索利用市场机制推动节能减排。落实部市土地管理制度改革合作协议，创新土地管理模式。

　　（七）扩大对内对外开放

　　大力实施"走出去"战略，继续推进中国-越南（深圳-海防）经贸合作区建设。鼓励本土企业更好地利用国际资源，在海外创建自有品牌、营销网络和研发中心。积极发展一般贸易进口，培育一批大宗商品、高端消费品的国际总代理、总经销企业。实施招商、招研、招才三大工程，集聚国内外优质资源。

　　充分用好计划单列市管理权限，突破体制机制障碍，加大开发建设力度，全力推进前海深港现代服务业合作区发展。继续推进河套地区规划研究工作，初步形成河套建议发展大纲图。全力推进东部通道、机场快速联络线等跨界基础设施建设，加强深港机场合作，促进形成无缝衔接、换乘便利的城际交通网络。加快引进香港高校来深合作办学。抓住ECFA实施机遇，加强与台湾经贸合作。

　　牵头编制深莞惠总体发展规划，加快三市边界地区合作开发研究。以观澜河、龙岗河、坪山河水环境综合整治工程为重点，加大跨界河流治理力度。实施跨界公交运营合作计划，打通市际断头路，促进交通等基础设施的全面对接。积极推动深中通道规划建设，强化与珠江西岸地区的联系。以建设深汕特别合作区为重点，全面推进与省内东西两翼和北部山区的产业合作。认真做好"双到"和对口支援工作。

　　（八）增进社会民生福利

　　稳定市场物价。保持对粮油肉菜等重点农产品价格的密切监测，做好价格异动应对预案，规范市场价格秩序。推进新一轮菜篮子工程建设，加强重要生活物资储备，确保市场供给。落实困难群众临时价格补贴政策，完善最低生活保障、失业保险标准与物价上涨挂钩的联动机制，重点解决好困难家庭和低收入者的生活保障问题。

　　增加居民收入。切实加大公共服务和民生领域财政投入，着力保基本、保基础、保基层，增进民生福利。积极争取参与国家收入分配制度改革试点，完善企业工资合理增长制度，逐步扩大中等收入者比重，着力提高低收入者的收入水平，力争居民人均可支配收入达到3.5万元。

　　促进充分就业。实施更加积极的就业政策，推进公共就业服务均等化，新增劳动力、失业人员、就业困难人员就业率达85%以上，零就业家庭户数保持动态归零。构建和谐劳动关系，逐步提高劳动合同和集体合同签订率。完善欠薪保障制度，切实维护劳动者合法权益。改善外来劳务工生产生活环境，帮助新生代劳务工更好地融入城市。

　　完善社会保障。健全地方补充养老保险、机关公务员和事业单位职员职业年金制度，完善社会保险制度和服务体系，继续扩大社会保险的覆盖面，稳步提高社会保险待遇。规范社保基金管理，力争实现保值增值。完善以最低生活保障制度为基础的综合性社会救助体系，建立贫困预警机制，实现低保对象和低收入困难群体救助全覆盖。大力发展慈善、老年人、妇女儿童和残疾人事业。

　　强化住房保障。全面落实住房公积金政策，深入实施人才安居工程。整合现有资源，拓宽保障性住房房源。年内多渠道筹措资金145亿元，落实建设用地126公顷，安排建设保障性住房6万套，新开工3万套，竣工1万套，对5000个户籍低收入住房困难家庭发放货币补贴。完善保障性住房配售机制，确保公平合理。加强房地产市场监管，打击投机炒作行为，促进房地产市场平稳健康发展。

　　优先发展教育。启动国家教育综合改革试点工作，积极创建广东省推进教育现代化先进市。推行统一的生均拨款制度，新增义务教育学位1.3万个、普高学位6500个。加强幼儿园规范化建设，确保80%的幼儿园达到规范园标准。支持清华、北大、哈工大深圳研究生院发展，加快深大西丽校区和医学院建设，推进南科大筹建工作，提高深职院、信息学院办学水平。发展职业教育和民办教育，办好特殊教育。

　　提高医疗水平。加快推进公立医院改革，启动公立医院联网运营。实施便民就医工程，构建分级诊疗、社区首诊、双向转诊的医疗服务模式。加快市第二人民医院内科住院大楼、北大医院外科住院大楼、新安医院、新明医院、宝荷医院等项目建设，滨海医院建成投入运营。引导优秀医务人员向社康中心合理流动，提高社康中心服务水平。支持中医药事业发展。

　　发展文化事业。加快当代艺术与城市规划展览馆等文化设施项目建设，推动公共文化体育设施免费开放，继续办好"深圳读书月"等公益文化活动，提高市民对城市的认同感和归属感。推进大鹏所城、客家民居等重要历史文化遗产保护工程，加强文物和非物质文化遗产保护。积极发展体育事业和体育产业，广泛开展全民健身运动。

　　改善公共交通。出台城市交通白皮书，做好地铁竣工道路恢复工作及公交接驳工程。采取综合措施治理出行难问题，加快打通断头路，改善片区交通出行环境，有效缓解梅林、布吉等关口交通拥堵。落实国家公交都市示范城市实施方案，加大公交线网开设和运力投放力度，力争全市公交分担率达50%以上。加强交通统筹管理，引导小汽车合理使用，鼓励市民出行选用公交工具。提倡绿色交通，逐步建成覆盖全市的现代步行系统。取消梧桐山隧道收费。

　　维护公共安全。构建治安立体防控体系，从严打击严重暴力犯罪活动，实现社会治安明显好转。规范管理虚拟社会，严厉打击网络违法犯罪行为。进一步完善应急机制，全面提升应急管理能力。加强安全生产监管，做好重点区域安全隐患排查整治工作，防范重特大事故发生。深入推进食品药品安全专项整治，确保市民饮食用药安全。健全信访工作机制，有效解决民生信访问题。加强人口管理和计生工作，控制人口过快增长。完善积分入户政策，逐步提高户籍人口比重。同时，扎实推进统计、司法、双拥、档案、民族、宗教、外事、港澳、台务、侨务、民防、气象、防震减灾等各项工作。

　　（九）强化政府自身建设

　　认真接受市人大及其常委会的法律监督和工作监督，坚持依法向市人大常委会报告工作。认真接受市政协的民主监督，加强与市政协的工作沟通和协商，主动听取各民主党派、人民团体和社会各界的意见和建议。切实执行市人大及其常委会通过的各项决议，及时办理人大代表建议和政协提案，提高办理工作的质量和效果。

　　充分利用大运会等重大活动压任务、打硬仗、练队伍，以时限倒逼进度，以目标倒逼责任，把工作压力传送到每个环节和岗位，激发干部队伍干事创业的干劲。全面推行政府绩效管理，加强政令检查和电子监察，完善奖惩制度，切实提高行政执行力。加强政府机关作风建设，倡导"比学赶帮超"，切实改进文风会风，推动政府工作提速提质提效。

　　以便利服务对象为导向，以深化政府投资项目审批制度改革为突破口，再造行政审批流程，实行跨部门串并联组合审批。深入推进政务公开，加大政府信息资源共享力度，完善行政服务大厅等窗口服务，提升政府服务水平。及时修改或废止不符合经济社会发展要求的规章和文件，实现行政服务事项法定化、标准化。

　　大力推进依法行政，提高行政执法水平，建设法治政府。完善重大事项集体决策、专家咨询、社会公示听证和决策效果评估制度，提高科学民主决策水平。加强政府内外各类监督，充分发挥监察、审计监督和媒体舆论监督的作用。加强对财政资金的监督管理和绩效评估，厉行节约，勤俭办事，用好纳税人的每一分钱。以制度建设为重点，加大从源头上防治腐败的工作力度，坚决查处腐败案件，保持惩治腐败的高压态势，推动廉洁城市建设。

　　各位代表，今年是中国共产党成立90周年，我们将发扬党的优秀传统，坚持党的群众路线，始终把群众利益放在首位，听民声、察民情、解民忧、纾民困，永远和人民群众在一起！我们将坚持民生为重、民事先办，以高度的责任感和使命感，在教育、医疗、文化、交通、住房、环保等领域，扎实办好一批看得见、摸得着的民生实事，让市民充分享受到经济发展的成果，向建党90周年献礼！

　　各位代表，"九万里风鹏正举"，深圳经济特区新的伟大事业已经启航。让我们高举中国特色社会主义伟大旗帜，坚持以邓小平理论和"三个代表"重要思想为指导，深入贯彻落实科学发展观，在市委的坚强领导下，解放思想，改革创新，求真务实，开拓进取，努力完成"十二五"的目标任务，创造"深圳质量"的新辉煌，为加快转型升级、建设幸福广东，为把深圳建设成为现代化国际化先进城市而努力奋斗！
